# Regulation of the human *LAT *gene by the Elf-1 transcription factor

**DOI:** 10.1186/1471-2199-7-4

**Published:** 2006-02-07

**Authors:** Timothy S Finco, Geri E Justice-Healy, Shivani J Patel, Victoria E Hamilton

**Affiliations:** 1 Department of Biology, Agnes Scott College, Decatur, GA 30030, USA

## Abstract

**Background:**

The *LAT *gene encodes an intracellular adaptor protein that links cell-surface receptor engagement to numerous downstream signalling events, and thereby plays an integral role in the function of cell types that express the gene, including T cells, mast cells, natural killer cells, and platelets. To date, the mechanisms responsible for the transcriptional regulation of this gene have not been investigated.

**Results:**

In this study we have mapped the transcriptional start sites for the human *LAT *gene and localized the 5' and 3' boundaries of the proximal promoter. We find that the promoter contains both positive and negative regulatory regions, and that two binding sites for the Ets family of transcription factors have a strong, positive effect on gene expression. Each site binds the Ets family member Elf-1, and overexpression of Elf-1 augments *LAT *promoter activity. The promoter also contains a Runx binding site adjacent to one of the Ets sites. This site, which is shown to bind Runx-1, has an inhibitory effect on gene expression. Finally, data is also presented indicating that the identified promoter may regulate cell-type specific expression.

**Conclusion:**

Collectively, these results provide the first insights into the transcriptional regulation of the *LAT *gene, including the discovery that the Ets transcription factor Elf-1 may play a central role in its expression.

## Background

The *LAT *gene encodes a 36–38 kD transmembrane protein that connects various cell surface receptors to downstream signaling events. Following receptor engagement, LAT becomes heavily tyrosine phosphorylated and subsequently binds a number of proteins containing SH2 domains, including members of the Grb2 family (Grb2, Gads, Grap) and phospholipase C-γ (PLC-γ) [1,2]. The interaction of Grb2, PLC-γ and other proteins with LAT facilitates their activation and leads to additional biochemical events required for a productive cellular response, including activation of the Ras-MAPK signaling pathway and an elevation in intracellular calcium levels [1,2].

One receptor type whose engagement results in LAT tyrosine phosphorylation is the T cell receptor (TCR) [3,4]. The essential role of LAT in TCR signaling has been revealed through the study of mutant Jurkat T cell lines that fail to express the *LAT *gene [5,6]. These LAT-deficient T cells display a number of signaling defects that prevent T cell activation and effector cell function. An analysis of LAT-deficient mice has also shown that the LAT protein is necessary for T cell maturation within the thymus [7]. In addition to its requirement for T cell development and function, LAT has important roles in other cell-types. For example, LAT is required for mast cell activation following engagement of the high affinity receptor for immunoglobulin E, for Natural Killer (NK) cell-mediated cytotoxicity, and for collagen, collagen-related peptide, and convulxin-mediated activation of platelets [8-12]. Moreover, engagement of the pre-B cell receptor on developing B cells induces LAT tyrosine phosphorylation, an event that may contribute to B cell differentiation [13-15].

The identified role of LAT in these cell-types is reflective of its expression pattern. *LAT *RNA and/or protein has been detected in the thymus, spleen, and peripheral blood cells but is absent in the colon, brain, small intestine, ovary, testis, prostate, kidney, skeletal muscle, and liver [3,4,16]. Examination of specific cell-types indicates that *LAT *gene expression is restricted to a subset of cell-types generated during hematopoiesis, namely T cells, mast cells, platelets, and NK cells [3,4,16]. *LAT *is also expressed at low levels during the early stages of B cell development and is subsequently down-regulated prior to B cell maturation [14,15].

To date, the means by which the *LAT *gene is transcriptionally regulated has not been investigated. In the present work, we have mapped the transcriptional start sites for the human *LAT *gene and localized the proximal promoter region to approximately 800 bp extending upstream of the translation start site. Within the promoter, we have identified a negative regulatory region and two Ets binding sites that are bound by the Elf-1 transcription factor. Mutation of these Ets sites significantly reduces promoter activity, and consistent with these findings, overexpression of Elf-1 potently enhances *LAT *promoter function. A Runx binding site is also identified that binds Runx-1, and which when mutated results in an increase in *LAT *promoter activity. The data presented also suggests that the identified promoter region may mediate cell-type specific expression of the *LAT *gene. Collectively, these results provide the first insights into the transcriptional regulation of the *LAT *gene, which encodes an essential adaptor protein linking cell-surface receptors to critical downstream signaling events.

## Results

### The *LAT *gene contains multiple transcription start sites

**Figure 1:**
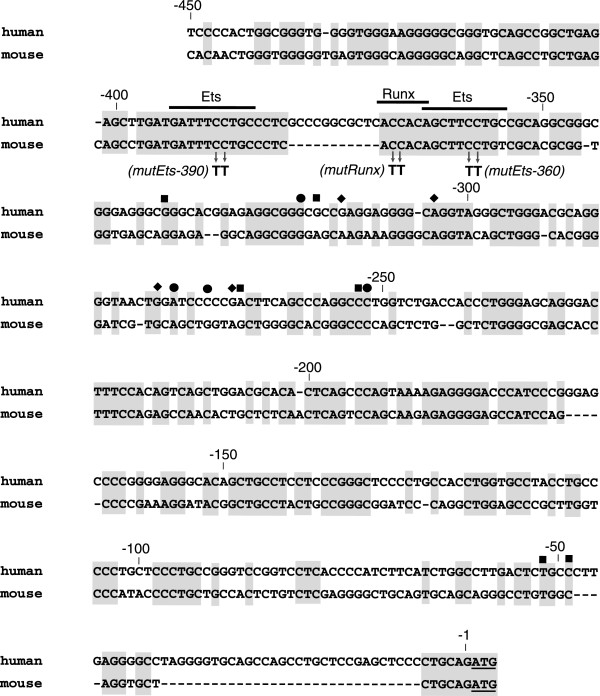
**A 450 bp region of the proximal *LAT *promoter**. The DNA sequence extending 450 bp upstream of the translation start site for the human gene and the homologous region from the mouse genome are shown. Bases conserved between the human and mouse are shaded. The 'A' nucleotide in the translation start site ATG codon (underlined) for the human sequence has been designated as +1 in this study. The transcription start sites identified for the endogenous *LAT *gene in Jurkat T cells have a '●' above the site, those mapped using RNA isolated from human thymus have a '■' above the site, and those that were identified in both Jurkat cells and thymus are indicated with a '◆' above the site. Potential binding sites for transcription factors of the Ets and Runx family are indicated with a bar above the site and the transcription factor name. Specific point mutations introduced into the -1000 to -1 promoter fragment are indicated below each site.

As an initial step in the identification and characterization of the *LAT *promoter, we mapped the transcription start site(s) for the human *LAT *gene using RNA ligase-mediated rapid amplification of cDNA ends (RLM-RACE) and poly(A) RNA isolated from Jurkat T cells or human thymus. The products of RLM-RACE, which are only derived from RNA transcripts extending to the terminal 5' cap structure [17,18], were ligated into the pBluescript^® ^vector and 15 of the resulting clones from each source of RNA were sequenced. The results indicate that the *LAT *gene possesses multiple transcription start sites that lie in a region spanning roughly 75 bp and located between 250 to 335 bp upstream of the translation start site (Fig. 1). The transcription start sites mapped to the same general region in Jurkat T cells and human thymus, and in many cases the same sites were utilized. Analysis of the DNA sequence surrounding the transcription start sites revealed that the region is G/C-rich but lacks consensus sites for either a TATA box or initiator elements. Similar to this situation, it has been observed that other G/C-rich promoters which lack a TATA box or initiator elements also possess multiple transcription start sites that span regions of 75 bp or more [19].

### The *LAT *promoter extends 800 bp upstream of the translation start site

**Figure 2:**
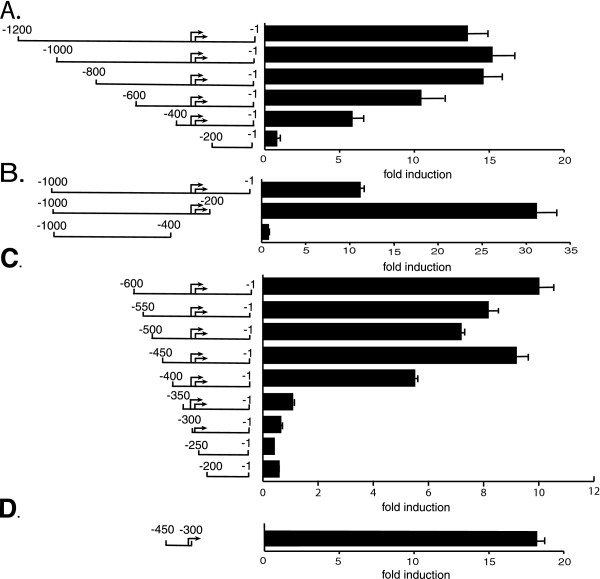
**Mapping the 5' and 3' boundaries of the *LAT *promoter. A.-D. **Jurkat T cells were transiently transfected with a pGL3-basic firefly luciferase reporter vector containing various DNA fragments extending upstream of the translation start site for the human *LAT *gene. Cells were also co-transfected with the internal control pRL-TK *Renilla *luciferase plasmid. Following incubation, cells were harvested, luciferase activity was quantitiated and after normalization of each sample based on *Renilla *luciferase activity, the fold activation relative to the empty pGL3-basic vector was calculated. Results presented are the average fold induction and the standard error of the mean (s.e.m.) from three or more independent experiments. For each graph, the human *LAT *promoter fragments inserted into pGL3-basic are shown schematically on the left. Arrows depict the two clusters of transcription start sites for the *LAT *gene.

To ascertain whether regions 5' and 3' of the transcription start sites possess promoter activity, a DNA fragment extending 1200 bp upstream from the translation start site was amplified by PCR and inserted adjacent to the firefly luciferase gene in the pGL3-basic vector. Of note, in this study the 'A' nucleotide of the translation start site ATG codon has been designated +1. Thus, the transcription start sites lie in the region from -335 to -250 and the 1200 bp fragment extends from -1200 to -1. Transient transfection into Jurkat T cells with the luciferase reporter construct containing the -1200 to -1 DNA fragment resulted in a greater than 10-fold increase in luciferase activity relative to the empty, promoterless pGL3-basic vector, demonstrating that this DNA fragment contains significant promoter activity (Fig. 2A). In order to more precisely map the boundaries of the promoter, sequential 5' and 3' deletions of 200 bp were introduced and the effect on promoter activity was determined in Jurkat cells. Deletion of the region from -1200 to -800 did not have an appreciable effect on promoter activity. However, deletion of the region from -800 to -600 resulted in a decrease in promoter activity, and a further decrease was observed when the region from -600 to -400 was removed. Finally, when the -400 to -200 region was deleted, promoter activity dropped to background levels (Fig. 2A).

Surprisingly, when the 3' 200 bp was removed from the -1000 to -1 promoter fragment (-1000 to -200), a substantial increase in promoter activity occurred (Fig. 2B). However, removal of an additional 200 bp from the 3' end (-1000 to -400) completely abrogated promoter activity (Fig. 2B). This latter result is most likely a consequence of eliminating all the transcription start sites (see Fig. 1). In summary, these results indicate that the proximal promoter for the *LAT *gene lies in a region extending from -800 to -1 relative to the translation start site, with the region from -800 to -200 having an overall stimulatory effect on promoter activity while the region from -200 to -1 has an inhibitory effect.

In this initial study of the *LAT *promoter, we chose to focus on the region spanning from -600 to -200 since it contained a significant portion of promoter activity (Fig. 2A). To more precisely map the positive regulatory elements within this region, a series of sequential 50 bp deletions from the 5' end of the -600 to -1 fragment were created and the resultant promoter activity of each DNA fragment was determined in Jurkat T cells. As shown in Fig. 2C, deletion of the region from -600 to -450 did not result in a significant loss in promoter function. In contrast, deletion of the region from -450 to -350 reduced promoter activity to background levels (Fig. 2C). These results indicate that the region spanning from -450 to -350 contains a substantial portion of the positive regulatory elements mediating transcription of the human *LAT *gene. Consistent with these data, a DNA fragment spanning from -450 to -300, which contains the -450 to -350 stimulatory region and a number of transcription start sites found between -350 to -300, displayed robust promoter activity (Fig. 2D).

### Linker scanning mutagenesis of the -450 to -350 region within the *LAT *promoter

**Figure 3:**
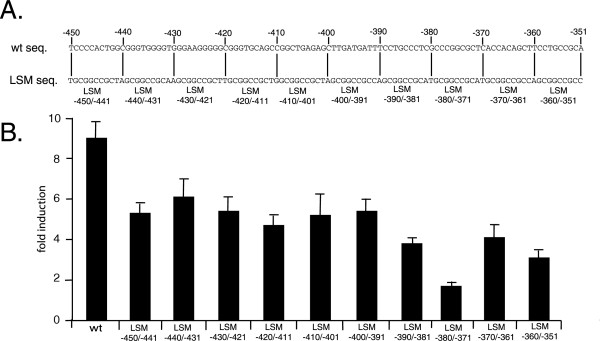
**Linker scanning mutagenesis of the -450 to -351 region of the *LAT *promoter. **Sequential 10 bp mutations were introduced throughout the -450 to -351 region in the context of the -1000 to -1 promoter fragment and the effects on promoter activity were determined in Jurkat T cells. **A**. The wild-type promoter sequence from -450 to -351 is shown on top. The lower strand depicts each 10 bp change, which incorporates a *Not I *restriction site (GCGGCCGC) plus one flanking nucleotide on each side. Base pair numbering is according to the translation start site, which was designated +1. **B**. Promoter activity for each linker scanning mutant was determined following transient transfection into Jurkat T cells. Following normalization of each sample based on the activity of co-transfected control pRL-TK plasmid encoding *Renilla *luciferase, the fold activation relative to empty pGL3-basic was calculated. The graph depicts the average fold activation and s.e.m. from three or more independent experiments.

Linker scanning mutagenesis throughout the -450 to -350 region, conducted by introducing sequential 10 bp mutations containing a *NotI *restriction site sequence, was performed with the -1000 to -1 promoter fragment and the effects on promoter activity were tested in Jurkat T cells (Fig. 3A and 3B). Each 10 bp mutation resulted in a decrease in promoter activity, with the most dramatic reduction occurring when the region from -390 to -350 was altered (Fig. 3B).

To ensure that the effects observed in the linker scanning mutagenesis experiments were not an artifact of the introduced sequence, we also constructed an additional set of linker scanning mutations throughout the -400 to -350 region, sequentially mutating the same 10 bp segments except introducing a sequence containing the *NheI *restriction site. Transient transfection of these reporter constructs in Jurkat T cells yielded results similar to those obtained in Fig. 3B except for the region spanning from -380 to -371, which when mutated in this latter set of experiments yielded only a ~50% decrease in promoter function (data not shown).

Collectively, the data from linker scanning mutagenesis indicates that the entire region from -450 to -350 is required for optimal *LAT *promoter activity in Jurkat T cells, with DNA sequences from -390 to -350 having the most prominent role.

### Two Ets sites are required for *LAT *promoter activity

Analysis of the -450 to -350 region using a variety of transcription factor binding site databases, including TRANSFAC^®^, JASPAR, and MatInspector [20-22], revealed a number of possible regulatory elements, including two sites that contain the core binding sequence (5'-TTCC-3') for the Ets family of transcription factors (see Fig. 1) [23-26]. These potential Ets sites, which lie within the critical -390 to -350 region, were of particular interest in light of the well-documented role Ets transcription factors play in the development and function of T cells and other cell types that express the *LAT *gene [24-26].

**Figure 4:**
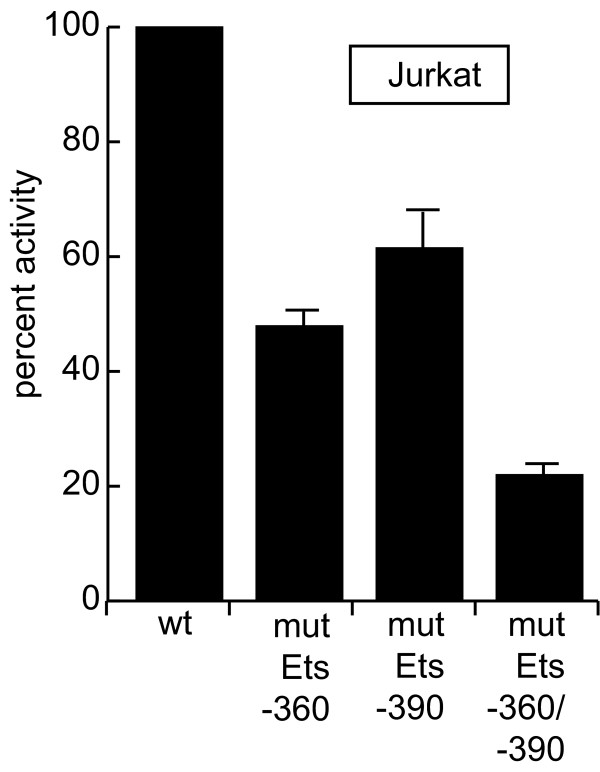
**Effect of mutating the Ets sites within the *LAT *promoter. **Site-directed mutagenesis was used to mutate the Ets site centered at -360 (mutEts -360), the Ets site centered at -390 (mutEts -390), or both Ets sites (mutEts -360/-390). Mutations were introduced into the -1000 to -1 promoter fragment within the pGL3-basic vector. Specific base pair changes are shown in Fig. 1. Reporter plasmids were transiently transfected into Jurkat T cells and fold activation was determined as previously described. The results depict the average fold induction and s.e.m. from three or more independent experiments.

To determine the function of each putative Ets site in the regulation of the *LAT *promoter, point mutations were introduced into each of these sites (see Fig. 1) in the context of the -1000 to -1 promoter fragment and the effect of each mutation was determined in Jurkat T cells. When either Ets site was mutated, a significant decrease in promoter activity was observed (Fig. 4). Furthermore, simultaneous deletion of both Ets sites resulted in a level of promoter activity less than when either site alone was mutated. These data demonstrate that both putative Ets sites have a strong, positive effect on *LAT *promoter function in T cells.

### Both Ets sites bind Elf-1, a member of the Ets transcription factor family

**Figure 5:**
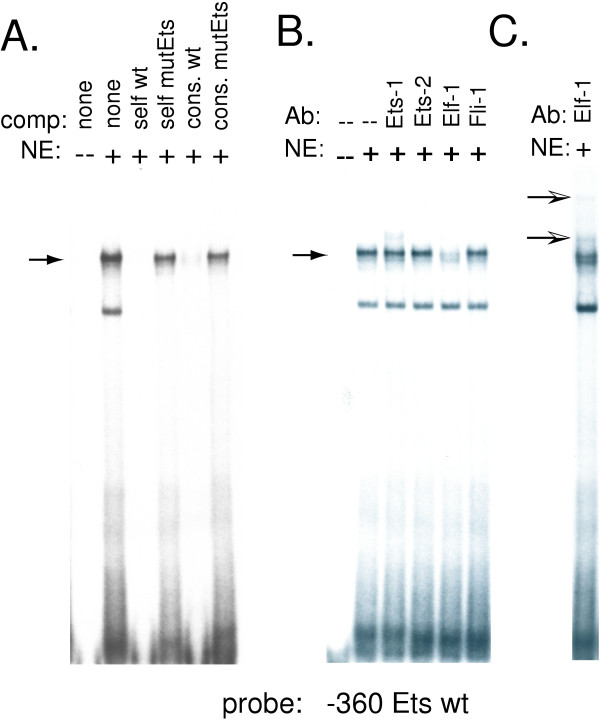
**The transcription factor Elf-1 binds to the -360 Ets site in the *LAT *promoter. A. **A DNA probe encompassing the Ets site centered at -360 (-360 Ets wt) was labeled with ^32^P and incubated with nuclear extracts from Jurkat T cells in the absence or presence of 100-fold molar excess of competitor DNA. Samples were subsequently loaded onto a nondenaturing polyacrylamide gel, electrophoresed, and then subjected to autoradiography. For competition experiments, the unlabeled competitor DNA was the same sequence as that of the probe (self wt), the same sequence as the probe except containing point mutations within the Ets site (self mutEts), the consensus sequence for Ets family members (cons. wt) or the consensus sequence except for point mutations within the Ets site (cons. mutEts). The arrow indicates the specifically bound complex. **B**. Experiments were conducted as in Fig. 5A except that the indicated antibody was added in place of competitor DNA. **C. **A longer exposure of the lane containing the Elf-1 antibody. The supershifted complexes are indicated with arrows.

Electrophoretic mobility shift assays (EMSAs) were used to identify protein complexes that specifically bind each of the putative Ets sites. ^32^P labeled DNA probes encompassing each Ets site were incubated with nuclear extracts from Jurkat T cells and then analyzed by nondenaturing polyacrylamide gel electrophoresis and autoradiography. Two major complexes were detected binding to the Ets site centered at -360 (Fig. 5A). One of these complexes was competed for with a 100-fold excess of unlabeled wild-type probe (self wt) but was not competed for when the Ets site was mutated (self mutEts), demonstrating that this complex specifically interacted with the Ets site. Of note, the mutant probe used in these competition experiments contained the same bp changes as those introduced into the Ets site in the promoter construct used in transient transfections.

Additional competition experiments were performed with an unlabeled probe containing a consensus Ets site (cons. wt) or the same probe except that the Ets site was disrupted by a point mutation (cons. mutEts) (Fig. 5A). The loss of binding in the presence of the consensus wild-type Ets sequence but not with the mutated sequence suggested that the specific complex contains members of the Ets family of transcription factors. In an effort to identify the particular Ets family members capable of binding the -360 Ets site, we performed antibody supershift experiments. The human genome contains 27 genes that encode members of the Ets family of transcription factors, 16 of which have been shown to be expressed in Jurkat cells [27]. We chose to focus on Ets-1, Ets-2, Elf-1, and Fli-1, Ets family members implicated in the regulation of gene expression in T cells and other cell types that express the *LAT *gene [23-26]. Although addition of antibodies to Ets-2 or Fli-1 had no discernible effect, in the presence of the Elf-1 antibody a significant decrease in the intensity of the specific complex was observed, and upon longer exposure of the gel, supershifted complexes were detected (Fig. 5B and 5C). It was also evident upon addition of the Elf-1 antibody that the specific complex contains at least two distinguishable bands, one of which appears unaffected by the Elf-1 antibody (Fig. 5B). Finally, in the presence of the Ets-1 antibody, a novel slower mobility band appeared. Although the appearance of this band was not accompanied by an appreciable decrease in the intensity of the specific complex, it is known that antibodies to Ets-1 can enhance its DNA binding capability, and thus we can not presently exclude the possibility that Ets-1 may also bind to this site in the LAT promoter. In summary, these results indicate that the -360 Ets site in the *LAT *promoter is capable of binding the Ets transcription factor Elf-1, and also possibly Ets-1.

**Figure 6:**
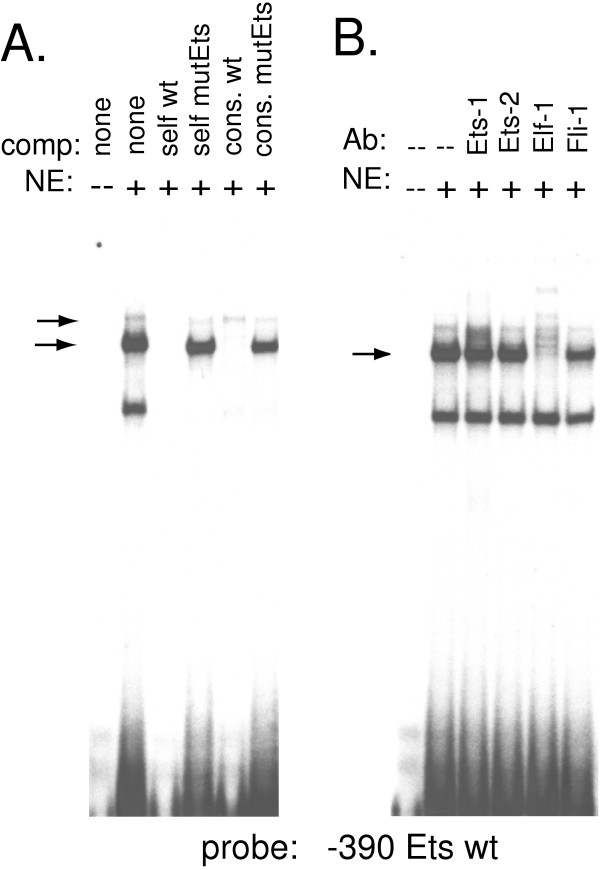
**Elf-1 interacts with the -390 Ets site. A. **A DNA probe encompassing the Ets site centered at -390 (-390 Ets wt) was labeled with ^32^P and incubated with nuclear extracts from Jurkat T cells in the absence or presence of 100-fold molar excess of competitor DNA. Samples were processed as described in the legend for Fig. 5. For competition experiments, the unlabeled competitor DNA was the same sequence as that of the probe (self wt), the same sequence as the probe except containing point mutations within the Ets site (self mutEts), the consensus sequence for Ets family members (cons. wt) or the consensus sequence except for point mutations within the Ets site (cons. mutEts). The arrows indicates the specifically bound complex. **B**. Experiments were conducted as in Fig. 5A except that the indicated antibody was added in place of competitor DNA.

When the Ets site centered at -390 was examined by EMSA, three complexes were detected (Fig. 6A). Two of these complexes were competed for with a 100-fold excess of unlabeled wild-type probe, but not when the Ets site was mutated, demonstrating that the binding of these complexes required an intact Ets site. When the consensus Ets site was used in competition experiments, the more prominent specific complex was competed for while the less prominent one was unaffected (Fig. 6A), suggesting that the more prominent complex contains Ets family members. The composition of the less prominent complex is uncertain at this time but may contain a transcription factor that binds to a site that overlaps the Ets site. In supershift experiments, addition of antibodies to Ets-1, Ets-2, and Fli-1 had no appreciable effect on the specific complexes, but when the Elf-1 antibody was added to the binding reaction an almost complete loss of the most prominent complex was observed, and this was accompanied by the appearance of slower mobility, supershifted bands (Fig. 6B). These data demonstrate that Elf-1 is capable of binding both Ets sites in the *LAT *promoter.

### Activation of the *LAT *promoter by overexpression of Elf-1

**Figure 7:**
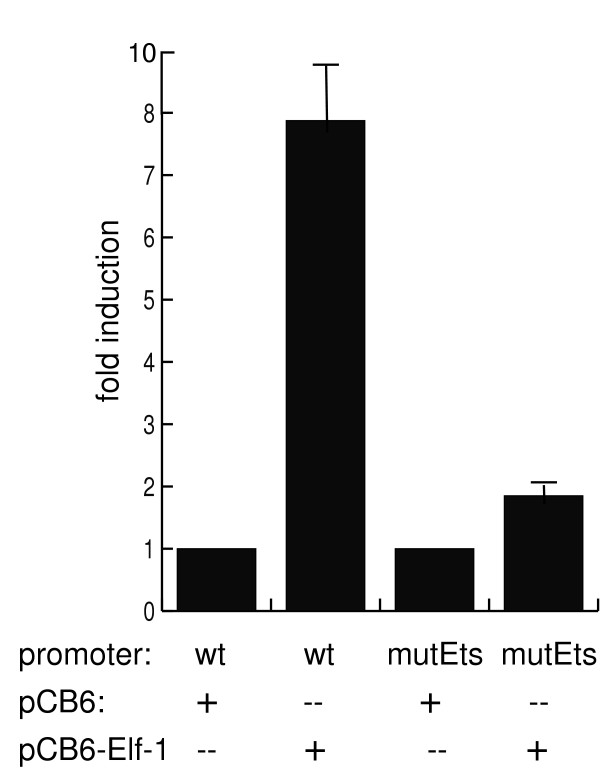
**Overexpression of Elf-1 stimulates *LAT *promoter activity. **The pGL3-basic reporter vector containing the -400 to -1 wt region (wt) of the *LAT *promoter or the -400 to -1 region with point mutations within both Ets sites (mutEts) were transiently transfected into HeLa cells in the presence of the parental pCB6 vector or the expression vector for Elf-1, pCB6-Elf-1. Twenty to twenty four hours later cells were harvested and fold activation was determined as described. The luciferase activity for each construct in the absence of co-transfected Elf-1 was then set at 1-fold. The resulted shown are the average fold induction and s.e.m. for at least three independent experiments. p < 0.05 for Elf-1 activation of the mutant promoter relative to that of wild-type as determined using the two-tailed *t *test.

To further explore the potential role of Elf-1 in the regulation of *LAT *gene expression, overexpression studies were conducted. Preliminary experiments were performed in Jurkat T cells and failed to reveal an effect of Elf-1 overexpression on *LAT *promoter activity (data not shown), a result likely attributable to the high endogenous levels of this transcription factor in these cells [27,28]. We therefore used HeLa cells for these experiments, which in contrast to Jurkat T cells express low to undetectable levels of the Elf-1 [27-29]. Overexpression of Elf-1 in HeLa cells resulted in a greater than 10-fold increase in LAT promoter activity (Fig. 7). Of note, identical results were obtained when either the -400 to -1 promoter fragment (Fig. 7) or the -1000 to -1 promoter fragment (data not shown) were used. To determine whether the observed activation by Elf-1 occurred through the Ets sites centered at -360 and -390, Elf-1 was overexpressed in the presence of the promoter construct containing point mutations in both these sites. Mutation of the Ets sites resulted in dramatic decrease in the ability of Elf-1 to stimulate promoter function (Fig. 7), results that clearly demonstrate Elf-1 can stimulate gene expression through both of the Ets sites in the *LAT *promoter.

### Role of the *LAT *promoter in cell-type specific expression

The *LAT *gene is expressed in T cells, NK cells, mast cells, platelets, and developing B cells but not in other cells types [3,4,16] To explore the potential role of the *LAT *promoter in mediating cell-type specific gene expression, we investigated promoter activity in various cell-types that either do or do not express the endogenous *LAT *gene. In addition to Jurkat T cells, the RBL-2H3 mast cell line has been shown to express the endogenous *LAT *gene while the HeLa epithelial cell line, the 70Z pre-B cell line, and the Raji mature B cell line do not [3,15]. Reporter vectors containing LAT promoter fragments of various lengths were transfected into each of these cell-types and following incubation the cells were harvested and promoter activity quantitated.

**Figure 8:**
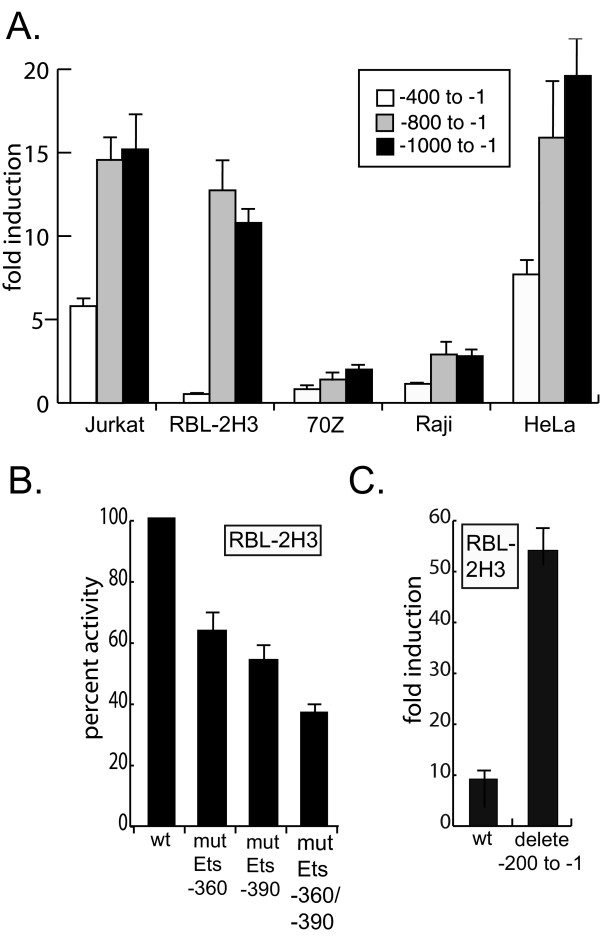
***LAT *promoter activity in various cell-types**. **A. **Reporter constructs containing the indicated regions of the human LAT promoter were transiently transfected into the indicated cell-types. Following incubation, samples were harvested and after normalization to *Renilla *luciferase activity fold activation was calculated relative to that obtained with the promoterless pGL3-basic vector which was set at 1-fold. **B. **RBL-2H3 cells were transiently transfected with either the wild-type -1000 to -1 reporter plasmid or reporter plasmids containing the -1000 to -1 fragment with the indicated site(s) mutated. Following incubation, samples were harvested and after normalization to *Renilla *luciferase activity, the activity obtained with the wild-type promoter was set at 100%. p < 0.05 for mutant reporters relative to wild-type using the two-tailed *t *test **C. **RBL-2H3 cells were transfected with the -1000 to -1 or -1000 to -200 reporter plasmids. Following incubation, samples were harversted, luciferase activities were measured and normalized to *Renilla *luciferase, and the activity obtained with empty pGL3-basic was set at 1-fold. For Fig. 8A-8C, the results shown are the average fold induction and s.e.m. from three or more independent experiments. For 8B and 8C, p < 0.05 for the mutant reporters relative to wild-type as determined using the two-tailed *t *test.

In addition to Jurkat cells, the -1000 to -1 *LAT *promoter fragment displayed significant activity in RBL-2H3 and HeLa cells but was relatively inactive in the 70Z and Raji B cell lines (fig. 8A). Experiments were also conducted in each cell line using the pGL3-control vector which contains the SV40 promoter and enhancer. The average fold activation for the pGL3-control vector was 50.1 for 70Z, 245.2 for Raji, 110.3 for RBL-2H3, and 300.6 for HeLa, indicating that the lack of appreciable *LAT *promoter function in 70Z and Raji cell lines is not a consequence of low transfection efficiencies. These data suggest that the lack of promoter activity in 70Z and Raji cells may explain why the endogenous *LAT *gene is not expressed in these cell-types. However, additional means of inhibiting *LAT *gene expression likely exist since the promoter retained activity HeLa cells, another cell-type that fails to express the endogenous *LAT *gene.

In contrast to results obtained in Jurkat T cells, a promoter fragment spanning from -400 to -1 was unable to support gene expression in RBL-2H3 cells (Fig. 8A). This result implies that this region of the promoter may operate differently in these two cells-types, and it was conceivable that the Ets sites located at -360 and -390 do not contribute to *LAT *promoter activity in mast cells. In order to determine the role of the Ets sites in mast cells, we tested the activity of the -1000 to -1 promoter fragment in the absence or presence of mutations in each site. Our results found that mutation of either Ets site caused a reduction in promoter activity, and a further decrease was seen when both Ets sites were mutated (Fig. 8B). Thus, the Ets sites centered at -360 and -390 are required for *LAT *promoter function in both Jurkat T cells and RBL-2H3 mast cells.

We also explored whether the inhibitory region spanning from -200 to -1 may be more active in mast cells since this could also explain why the -400 to -1 region was unable to support gene expression in RBL-2H3 cells. When the -1000 to -200 promoter construct was transfected into RBL-2H3 cells, promoter activity increased over 6-fold relative to the -1000 to -1 construct (Fig. 8C). When similar experiments were conducted in Jurkat cells, an approximately 3-fold increase was observed (Fig. 2B). Thus, the greater suppressive effect of the -200 to -1 region on *LAT *promoter activity in RBL-2H3 cells may provide one explanation why the -400 to -1 region lacks activity in this cell line.

### The *LAT *promoter contains a potential Runx binding site adjacent to the Ets site centered at -360

Alignment of the human *LAT *promoter sequence with the region upstream of the translation start site for the mouse *LAT *gene revealed that the Ets sites are 100% conserved between the two species. Interestingly, sequences adjacent to the Ets sites are also conserved, and close inspection of the region 5' of the Ets site centered at -360 revealed a potential binding site for the Runx family of transcription factors. The Runx family, also called CBFα, AML, or PEBP2, consists of three members: Runx-1, -2, and -3 [30,31]. These transcription factors contain a conserved 128 amino acid DNA binding domain that interacts with the consensus sequence 5'-ACCPuCA-3', and they can either activate or repress gene expression depending on the context of the specific promoter or enhancer [30,31]. Interestingly, in a number of instances Runx and Ets binding sites have been found adjacent to one another in gene regulatory regions. Moreover, it has been shown that one Runx family member, Runx-1, can physically interact with certain Ets proteins, resulting in cooperative DNA binding and thereby facilitating the activation of gene expression [30,32-38]. It was therefore conceivable that in a manner similar to other regulatory regions, Runx-1 and Ets proteins cooperatively interact at the *LAT *promoter to activate gene expression.

**Figure 9:**
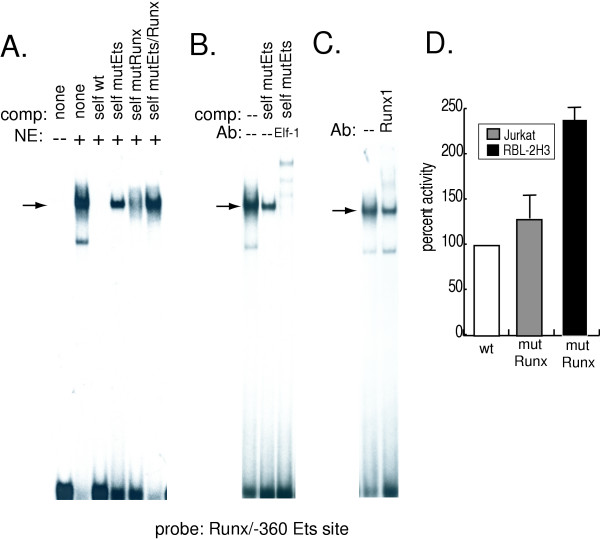
**The transcription factor Runx1 binds to the *LAT *promoter. A. **A DNA probe encompassing the Runx site and the Ets site centered at -360 (Runx/-360 Ets wt) was labeled with ^32^P and incubated with nuclear extracts from Jurkat T cells in the absence or presence of 100-fold molar excess of unlabeled competitor DNA. Samples were processed as described in the legend for Fig. 5. For competition experiments, the unlabeled competitor DNA was the same sequence as that of the labelled probe (self wt), the same sequence as the probe except containing point mutations within the Ets site (self mutEts), the Runx site (self mutRunx) or both sites (self mutEts/Runx). **B. **Experiments were conducted as in Fig. 9A, with the exception that the Elf-1 antibody was also added to the indicated sample. **C. **Experiments were conducted as described in Fig. 9A except that the Runx-1 antibody was added instead of unlabeled competitor. For A-C, the arrow denotes specific complexes. **D**. Jurkat and RBL-2H3 cells were transfected with the wild type -1000 to -1 reporter plasmid or the -1000 to -1 reporter plasmid with a mutation within the Runx site. Following incubation, samples were harversted, luciferase activities were measured and normalized to *Renilla *luciferase, and the activity obtained with empty pGL3-basic was set at 1-fold. The results shown are the average fold induction and s.e.m. from three or more independent experiments. p < 0.05 for the mutant reporters relative to wild-type as determined using the two-tailed *t *test.

As a first step in testing this possibility, EMSA was used to determine if Runx-1 could bind to the putative Runx site in the *LAT *promoter. In these experiments a ^32^P labeled probe encompassing both the Runx site and the adjacent Ets site at -360 was used, in contrast to results shown in Fig. 5 in which case the ^32^P labelled probe contained the Ets site but not the Runx site. In the presence of Jurkat nuclear extracts, a complex formed that contained a diffuse band that extended above and below a more defined central band (Fig. 9A). Both the diffuse and central band were effectively competed for by an excess of unlabeled wild-type probe. When the unlabeled competitor contained a mutation in the Ets site, the diffuse band was competed for but the more defined central band was not. Conversely, when the unlabeled competitor contained a mutation in the Runx site, the central band was competed for but not the more diffuse band. (Fig. 8A). Finally, when the unlabeled competitor contained a mutation in both the Runx and Ets sites, no bands were competed. Taken together, these results demonstrate that the diffuse band represents proteins specifically interacting with the Runx site while the more defined central band represents proteins specifically binding to the Ets site. Of note, the prominent central band that remained in the presence of the competitor containing a mutation in the Ets site could be supershifted with an antibody to Elf-1 (Fig. 9B), which is consistent with our earlier data identifying Elf-1 as the major Ets family member binding to this site.

To determine whether the diffuse band may contain Runx-1, the member of the Runx family shown to cooperate with Ets proteins, we performed supershift assays. The addition of the Runx-1 antibody to the binding reaction caused a loss of the diffuse band and the appearance of a supershifted complex (Fig. 9C). Thus, the identified Runx site in the *LAT *promoter is capable of binding Runx-1. To investigate the role of the Runx site in *LAT *promoter function, we introduced a point mutation within the putative Runx site in the context of the -1000 to -1 promoter construct and tested its activity in both Jurkat and RBL-2H3 cells. In contrast to our expectations, mutation of the Runx site resulted in an increase in *LAT *promoter function in both Jurkat and RBL-2H3 cells (Fig. 9D), implying that this site has an inhibitory effect on *LAT *promoter function.

## Discussion

This is the first study to examine the mechanisms regulating transcription of the human *LAT *gene. The data presented indicates that the gene contains numerous transcription start sites and that the proximal promoter extends approximately 800 bp upstream of the translation start site. Within the promoter we identified two Ets sites which when mutated dramatically reduced transcriptional activity. Both Ets sites were shown to bind Elf-1, and consistent with this finding, promoter activity was significantly augmented following Elf-1 overexpression. We also identified a binding site for Runx-1 that appears to suppress promoter activity. Finally, the absence of appreciable *LAT *promoter activity in 70Z and Raji B cell lines may contribute to the cell-type specific expression of this gene.

Our results demonstrate that deletion of the region from -200 to -1 leads to a 3-fold or greater increase in *LAT *promoter activity. Since this region lies downstream of the transcription start sites, the effects observed could conceivably be mediated at the level of transcription or a subsequent step in the gene expression process, such as mRNA stability or the efficiency of translation. However, this region still possesses inhibitory activity when moved upstream of the transcription start sites in the *LAT *promoter (data not shown), suggesting that the effect occurs at the level of transcription. Although the biological relevance of the inhibitory region is presently unknown, the fact that the magnitude of its activity varies between Jurkat and RBL-2H3 cells suggests that it may modulate cell type specific expression levels for the *LAT *gene.

Deletion analysis and linker scanning mutagenesis revealed that the region from -390 to -350 was particularly important for *LAT *promoter function. Within this region we identified two Ets sites and demonstrated that these sites bind the Ets transcription factor Elf-1. The finding that the *LAT *gene is regulated by Elf-1 is consistent with the known functions of Elf-1 and other members of this transcription factor family. Ets proteins have been shown to play a critical role in the development and function of T cells as well as other cell types of the immune system [25,26], and Elf-1 has been implicated in the regulation of a number of genes expressed in cells of hematopoietic origin, including genes for the TCRζ chain, the CD4 co-receptor, the immunoglobulin heavy chain, the cytokine IL-2, and CD1D1 molecule [39-42]. The fact that both Ets sites are 100% conserved in human, mouse, and rat (Fig. 1 and data not shown) also suggests that these sites play a fundamental role in the regulation of *LAT *gene expression.

Adjacent to the Ets site centered at -360 we identified a Runx site that is capable of binding Runx-1. Like the Ets sites, this Runx site is 100% conserved in human, mouse, and rat (Fig. 1 and data not shown). Similar to Ets, Runx family members have been shown to be critical for hematopoiesis and the expression of hematopoietic genes, where they positively or negatively regulate gene expression depending on the context of their binding site and/or prevailing intracellular conditions [30,31,43]. For example, during T cell development and/or in mature T cells, Runx family members positively regulate expression of the interleukin-3, granulocyte macrophage-colony stimulating factor, and TCR α and β genes [44-47] while they inhibit expression of the CD4 gene [48].

A number of genes contain promoter or enhancer elements with adjacent Ets and Runx binding sites, including the TCR α and β enhancers, the osteopontin promoter, the immunoglobulin μ heavy chain enhancer, and the enhancers of the polyomavirus and the Moloney leukemia virus [33,34,49-52]. Both Runx and Ets proteins contain intramolecular inhibitory domains that can impede DNA binding and studies have shown that Runx-1 and certain members of the Ets family, namely Ets-1, MEF, and NERF [32,34,36-38,53], can interact with each other, displacing the inhibitory domains and thereby stimulating DNA binding and transactivation. Elf-1 has also been shown, at least *in vitro*, to be capable of interacting with Runx-1 [32]. Based on these findings, and the close proximity of the Ets and Runx sites within the *LAT *promoter, we hypothesized that the Runx site would behave similarly to the Ets site and contribute to the activation of *LAT *gene expression. However, our results revealed that the Runx site actually has a repressive effect on LAT promoter activity, a result analogous to the inhibitory role of Runx sites in the CD4 gene silencer region [30]. It is possible that Runx-1 does not interact with Elf-1 at the *LAT *promoter and thus no cooperative effects would be expected. Consistent with this possibility, our EMSA data demonstrate that Elf-1 and Runx-1 can independently bind their respective sites in the *LAT *promoter (Fig. 9). Runx proteins also interact with a number of other proteins that influence whether they have a positive or negative effect on gene expression [30,31,43]. Thus, Runx-1 may recruit to the *LAT *promoter proteins that actively inhibit gene expression. It is also possible that under different circumstances, for example during hematopoiesis or in other cell-types, members of the Runx and Ets families cooperatively bind to the adjacent sites in the *LAT *promoter to facilitate gene expression.

Our data from the analysis of *LAT *promoter activity in various cell-types is intriguing. The lack of significant *LAT *promoter activity in 70Z and Raji B cells, which do not express the endogenous *LAT *gene, suggests that the promoter region identified in this study may mediate cell-type specific expression of this gene. However, other mechanisms are likely to be involved in cell-type specific expression since the *LAT *promoter retains substantial activity in HeLa cells, another cell line that fails to express the endogenous gene [3]. In this latter situation, it is conceivable that chromatin structure may play a more predominate role in cell-type specific regulation. It should also be noted that since Elf-1 is expressed in B cells and has been shown to activate gene expression in B cells [39,54], it is unlikely that this transcription factor alone plays a central role cell-type specific expression of *LAT*.

The results analyzing *LAT *promoter function in Jurkat and RBL-2H3 cell lines, both of which express the endogenous *LAT *gene [3], indicates that functional differences exist. In particular, a promoter fragment spanning from -400 to -1 fails to support gene expression in the RBL-2H3 mast cell line. One possible explanation of this result is that the Ets sites do not exert a positive effect on gene expression in mast cells. However, our data indicates that this is not the case and instead points to another explanation, namely that the -200 to -1 region and the Runx sites have a greater suppressive effect in mast cells, and thereby may mitigate any positive contribution made by factors binding to the Ets sites. The results in RBL-2H3, Jurkat, and HeLa cells also indicate that additional regulatory elements exist in the region upstream of -400, and in particular in the region spanning from -450 to -400. We have identified a number of potential Sp-1 binding sites within this section of the promoter and are currently exploring the role these sites may play in *LAT *gene regulation (data not shown). This and additional research will further elucidate the role of various transcription factors in the regulation of *LAT *gene expression, and should provide further insights into the role each of these factors play in the cell-type specific expression of this gene.

## Conclusion

The goal of this study was to investigate the mechanisms regulating transcription of the *LAT *gene, which is expressed in a number cell-types generated during hematopoiesis. We have defined the proximal promoter region and found binding sites for two families of transcription factors, Runx and Ets, which are known to play important roles in the development and function of hematopoietic cells. These sites are capable of binding Runx-1 and Elf-1 and appear to have opposing effects on *LAT *promoter function. These results provide initial insights into the regulation of the *LAT *gene and provide a framework for further research.

## Methods

### Cell lines

All cell lines were obtained from the American Type Culture Collection. The Jurkat human acute Leukemia T cell line, the 70Z/3.12 (70Z) mouse pre-B cell line, and the Raji human lymphoblast-like B cell line were each grown in RPMI-1640 media. The rat RBL-2H3 mast cell line and the human HeLa epithelial cell line were grown in DMEM media. All media was supplemented with 10% fetal calf serum, penicillin, and streptomycin. The 70Z cell media also contained 50 μM β-mercaptoethanol.

### Plasmids

A pBAC plasmid containing the human LAT gene and the surrounding genomic region was digested with *NcoI *and a 3543 bp DNA fragment extending approximately 3200 bp upstream of the *LAT *translation start site was subcloned into pBluescript KS II+ (Stratagene). This plasmid and primers incorporating flanking *KpnI *and *NheI *restriction sites were used in PCR to generate putative promoter fragments that were then subcloned into *KpnI *and *NheI *digested pGL3-Basic plasmid (Promega Corp.). This placed the putative LAT promoter fragments immediately upstream of the firefly luciferase gene in pGL3-Basic. For linker scanning mutagenesis, sequential 10 bp mutations incorporating a *NotI *restriction site were constructed as follows: two fragments of the promoter region were generated by PCR. For one fragment (*KpnI*-*NotI*), the sense primer at -1000 contained a flanking *KpnI *restriction site while the anti-sense primer contained a *NotI *site spanning the region to be mutated. For the second fragment, the sense primer contained the *NotI *site and the anti-sense primer at -1 contained a flanking *NheI *site. PCR products were purified, then digested with *KpnI *and *NotI *or *NotI *and *NheI*, and finally cloned into *KpnI *and *NheI *digested pGL3-Basic.

Site-directed mutatgenesis of the putative Ets and Runx DNA binding sites was conducted using the QuikChange^® ^II Site-Directed Mutagenesis kit (Stratagene). The specific base pair changes were as follows: The Ets site centered at -390 was changed from GATTTCCTGC to GATTT**TT**TGC, the Runx site at -365 was changed from ACCACA to A**TT**ACA, and the Ets site centered at -360 was changed from AGCTTCCTGC to AGCTT**TT**TGC. All plasmids created for these studies were sequenced to ensure that unwanted mutations were not incorporated during their construction.

The internal control pRL-TK *Renilla *lucifierase vector and the pGL3-Control vectors were purchased from Promega Corp. The expression vectors pCB6-Elf-1, along with the parental pCB6 vector, were provided by Dr. Scott W. Hiebert, Vanderbilt University School of Medicine, Nashville, Tennessee.

### RLM-RACE

RNase Ligase-mediated Rapid Amplification of cDNA ends (RLM-RACE) was performed using the FirstChoice^® ^RLM-RACE kit (Ambion, Inc). In this procedure, Poly(A) RNA was isolated from Jurkat T cells using the PolyATtract mRNA isolation System III (Promega Corp) and treated with calf intestinal phosphatase to remove the 5' phosphate from truncated mRNA species. Tobacco acid pyrophosphatase treatment then removed the 5' cap structure from full length RNA, followed by ligation of the 5' RACE adaptor. Reverse transcription followed by two rounds of PCR resulted in amplification of the 5' portion of the *LAT *cDNA. PCR products were separated by agarose gel electrophoresis, gel-purified, and cloned into pBluescript II KS+ for DNA sequencing. A similar series of steps were used to map the *LAT *transcription start sites using Human Thymus RACE-Ready cDNA (Ambion, Inc.).

### Transient transfection and Luciferase assay

Jurkat, RBL-2H3, and Raji cells were transiently transfected by electroporation at 950 μF and either 240 V (Jurkat, Raji) or 300 V (RBL-2H3) using 1-5 × 10^6 ^cells, 2.5 μg of pGL3 firefly luciferase reporter plasmid and 0.25 μg of the internal control phRG-TK *Renilla *luciferase plasmid per sample. HeLa cells were also transfected by electroporation as described above except for experiments involving the overexpression of Elf-1, in which case 4 μg of the parental pCB6 vector or Elf-1 expressing pCB6-Elf-1 was included. For all tranfections, pBluescript II KS+ plasmid DNA was also added to bring the final amount of DNA to 10 μg. 70Z cells were transfected using 2.5 × 10^5 ^cells, 0.9 μg of pGL3 reporter plasmid, 0.1 μg of phRG-TK plasmid, and 3 μl of FUGENE 6 reagent (Roche Applied Science) per sample according to the manufacturer's protocol. Following transfection, cells were resuspended in their respective media, incubated 20–24 hr, then harvested and assayed for firefly and *Renilla *luciferase activity using the Dual-Luciferase Reporter Assay System (Promega Corporation). The ratio of firefly to *Renilla *luciferase activity was calculated for each sample and the value obtained using the promoterless pGL3-basic vector was set at one-fold activation except for Elf-1 overexpression studies in HeLa cells. In these experiments, the ratio of firefly to *Renilla *luciferase activity obtained with the wild-type or mutEts *LAT *reporter constructs and pCB6 was set at one-fold activation.

Duplicate or triplicate samples were included for all transfections and the average and standard error of the mean (s.e.m.) for each reporter plasmid or condition was calculated from three or more independent transfection experiments using at least two preparations of each test plasmid.

### Preparation of nuclear extracts and the electrophoretic mobility shift assay

For the preparation of nuclear extracts, Jurkat T cells were washed once with PBS, then lysed in a buffer containing 10 mM HEPES, pH 7.6, 60 mM KCl, 1 mM EDTA, 0.075% NP-40, 1 mM DTT, 1 mM PMSF, 5 μg/ml each of aprotinin, leupeptin, and pepstatin. The resulting lysate was centrifuged at 1500 rpm for 4 min and the nuclear pellet washed once with lysis buffer lacking NP-40. The nuclear pellet was then resuspended in nuclear extract buffer (20 mM Tris, pH 8.0, 400 mM NaCl, 1.5 mM MgCl_2_, 0.2 mM EDTA, 0.5 mM PMSF, and 20% glycerol) and incubated on ice for 10 min. Centrifugation at 14 K rpm for 15 min followed, and the protein concentration in the resulting supernatant (nuclear extract) was determined by the Bio-Rad DC Protein Assay prior to storage at -80°C.

The ^32^P labeled probes used in the electrophoretic gel mobility shift assay (EMSA) were prepared by filling in annealed oligonucleotides with the Klenow fragment of DNA polymerase in the presence of [α-^32^P]dCTP and other dNTPs followed by purification using NucTrap probe purification columns (Stratagene). The sequence of the probes used in EMSAs are as follows (mutations are underlined): -390 wt : 5'-GCTTGATGATTTCCTGCCCTCGCC-3'; -390 mut Ets: 5;-GCTTGATGATTTTTTGCCCTCGCC-3'; -360 wt: 5'-GGCACAGCTTCCTGCCGCAGGCC-3'; -360 mut Ets: 5'-GGCACAGCTTTTTGCCGCAGGCC-3'; Runx/-360 Ets wt: 5'-GCGCTCACCACAGCTTCCTGCCGCAGG-3'; Runx/-360Ets mutRunx: 5'-GCGCTCATTACAGCTTCCTGCCGCAGG-3'; Runx/-360Ets mutEts: 5'-GCGCTCACCACAGCTTTTTGCCGCAGG-3'; Runx/-360Ets mutEts/Runx: 5'-GCGCTCATTACAGCTTTTTGCCGCAGG-3'; consensus Ets: 5'-GATCTCGAGCAGGAAGTTCGA-3'; consensus mutEts: 5'-GATCTCGAGCAAGAAGTTCGA-3'. Note that the GG at the 5' end of the -360 Ets probe and the terminal C nucleotide at the 3' end were added to aid in ^32^P labeling.

For EMSAs, 5 μg of nuclear extract was used in DNA binding reactions containing 10 mM HEPES, pH 7.6, 50 mM KCl, 1 mM EDTA, 1 mM DTT, 5% glycerol, and 0.2 μg sheared salmon sperm DNA. For competition experiments a 100-fold molar excess of unlabeled competitor DNA was also added, while for supershift experiments 1 μl of antibody was used. The Ets-1 (C-20), Ets-2 (H-140), Elf-1 (C-20), Fli-1 (C-19), and Runx1 (N-20) antibodies used in supershift experiments were purchased from Santa Cruz Biotechnology, Inc. Samples were incubated on ice for 15 min, 20 K cpm of labeled probe was then added, and samples incubated an additional 30 min prior to loading onto a 4.5% nondenaturing polyacrylamide gel. Samples were electrophoresed at 4°C and 20 mA using a 0.5× TBE running buffer. After the separation of complexes, the gel was dried and exposed overnight to Kodak Biomax film at -80°C using an intensifying screen.

### Accession numbers

The accession number for the human, mouse and rat DNA sequences are: [GenBank:AC109460](human); [EMBL: AJ438435](mouse); [EMBL: AJ001184] (rat).

## Authors' contributions

TF conceived the study, its design and coordination, and wrote the manuscript. TF, GJ, SP, and VH created the various reporter constructs and conducted the transient transfection experiments. TF and GJ carried the EMSA studies, and TF performed the RLM-RACE experiments.
